# Patterns of Bodyweight Changes in Patients With Hypothyroidism, a Retrospective Study From Basrah, Southern Iraq

**DOI:** 10.7759/cureus.15408

**Published:** 2021-06-02

**Authors:** Haider A Alidrisi, Samih A Odhaib, Mahmood Thamer Altemimi, Abbas A Mansour

**Affiliations:** 1 Diabetes and Endocrinology, Faiha Specialized Diabetes, Endocrine and Metabolism Center, Basrah, IRQ; 2 Endocrinology, College of Medicine, University of Basrah, Basrah, IRQ; 3 Diabetes and Endocrinology, Thi Qar Specialized Diabetes, Endocrine and Metabolism Center, Thi Qar Health Directorate, Basrah, IRQ; 4 Medicine, College of Medicine, University of Basrah, Basrah, IRQ

**Keywords:** hypothyroidism, bodyweight, weight gain, levothyroxine, thyroid-stimulating hormone

## Abstract

Background

Weight gain is one of the most important hypothyroidism-related concerns in patients with hypothyroidism. However, unexpectedly, levothyroxine replacement does not necessarily result in body weight (BWT) reduction among those patients. The study aimed to assess the patterns of BWT changes through time in patients with hypothyroidism.

Method

In a retrospective database study from Faiha Specialized Diabetes, Endocrine, and Metabolism Center, a total of 346 adult patients with hypothyroidism (192 newly diagnosed and 154 known hypothyroidism patients) who had one visit every three months, five visits in one year were included. Of these, 116 new and 69 known hypothyroidism patients had completed nine visits in two years. Each visit involved thyroid-stimulating hormone (TSH) and BWT measurements. Patients with chronic liver or renal disease, diabetes mellitus, thyroid cancer, or other malignancies, pregnancy, and steroid or hormonal therapies were excluded. The patients were further subdivided based on average TSH levels into controlled (TSH ≤ 4.2 μIU/ml) and uncontrolled (TSH > 4.2 μIU/ml). Repeated measures analysis of variance (ANOVA) with a Greenhouse-Geisser correction and post hoc tests using the Bonferroni correction were used to evaluate TSH and BWT changes through the study.

Results

Both in newly diagnosed and known hypothyroidism patients with an average TSH > 4.2 μIU/mL, BWT increased significantly through visits over one and two years. For newly diagnosed patients assessed over one year (F(2.41, 321.60) = 3.28, p = 0.03), the mean BWT increase was 1.4 ± 0.38 kg from 3rd to 12th month visits (p = 0.004). For newly diagnosed patients assessed over two years (F(3.10, 263.89) = 9.08, P < 0.0005), the mean BWT increase was 3.02 ± 0.77 kg from 3rd to 24th month visits (p = 0.007). For patients with known hypothyroidism assessed over one year (F(2.56, 187.47) = 7.11, p = 0.0003), the mean BWT increase was 1.97 ± 0.64 kg at 12th month visit, and over two years (F(2.35, 77.56) = 4.67, P = 0.009), the mean BWT increase was 3.78 ± 1.26 kg at 24th month visit. While in all other patients with an average TSH ≤ 4.2 μIU/mL, the BWT changed non-significantly through the visits for newly diagnosed patients over one year and two years (p = 0.10, 0.34*,* respectively), and known patients over one year and two years (p = 0.47, 0.34, respectively).

Conclusion

Contrary to what is believed, adequate treatment with levothyroxine does not associate with weight reduction. Instead, either the patient kept on the same weight or continued to gain more weight.

## Introduction

The relationships between body weight (BWT) and thyroid status are complex. Thyroid hormones play an important role in BWT regulation, mainly through regulating energy expenditure [1,2]. It is well established that thyroid dysfunction, including hyperthyroidism and hypothyroidism, leads to significant changes in BWT and resting metabolic rate (RMR) [1,3].

Patients with overt hypothyroidism often present with an obvious BWT gain and those with some BWT loss after starting levothyroxine (LT4) therapy. The degree of BWT change with thyroid dysfunction and the effect of treatment on BWT are surprisingly poorly understood. It is challenging to evaluate thyroid hormones in relation to BWT change in observational studies because the causes of BWT change are heterogeneous and often not well clear. In addition, few studies have examined RMR, a factor associated with both thyroid function and energy expenditure [1,4], in relation to thyroid hormones during BWT change. However, following LT4 treatment for overt hypothyroidism, BWT loss appears to be modest and mediated primarily by loss of water rather than fat [5].

This study aimed to evaluate the pattern of BWT changes in patients with hypothyroidism following the initiation of LT4 therapy in Basrah. This article was previously presented as a meeting abstract at ENDO 2021, the annual meeting of the Endocrine Society, held virtually on March 20-23, 2021.

## Materials and methods

In a retrospective database study at Faiha Specialized Diabetes, Endocrine, and Metabolism Center (FDEMC) in Basrah, Southern Iraq, we assessed patients with primary hypothyroidism, aged 18 years or older, regularly attending the center for follow-up from 2008 to 2019. Using the inclusion criteria, we selected patients who had completed at least five visits over one year (one visit every three months) and, from them, those who had completed at least nine visits over two years (one visit every three months). Each visit should have included measurements of thyroid-stimulating hormone (TSH) and BWT.

**Figure 1:**
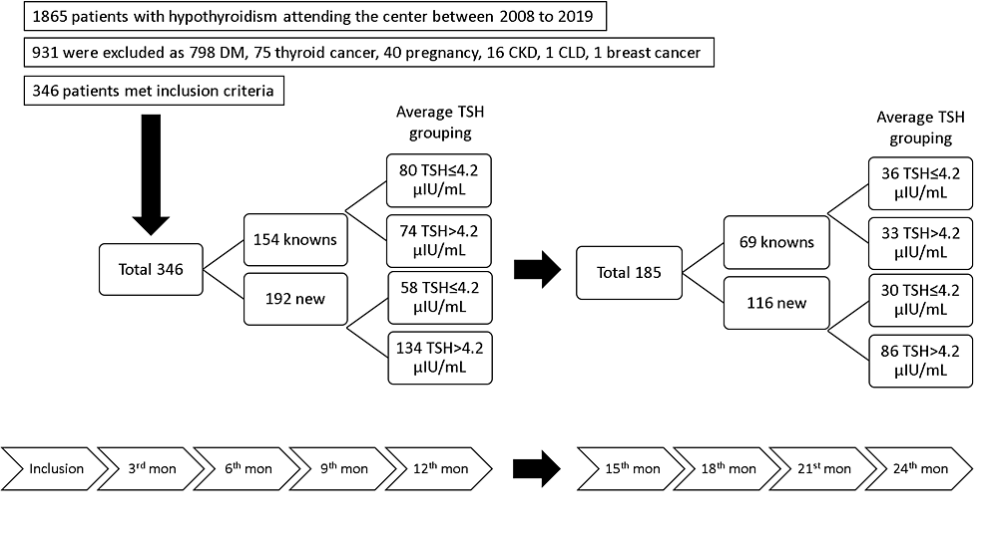
Flowchart of the evaluation of the patients with hypothyroidism included in the study. DM, diabetes mellitus; CKD, chronic kidney disease; CLD, chronic liver disease; TSH, thyroid-stimulating hormone

To compare the effects of recent and LT4 treatment, the patients were classified as “new” and “known” hypothyroidism. The “new” were patients diagnosed and started LT4 treatment in the center, while “known” were patients already been diagnosed and on LT4 treatment for more than a year but referred to the center for LT4 treatment adjustments, as shown in Figure 1. As the result of the expected possibility of their effects on BWTs, chronic liver disease (CLD) or chronic kidney disease (CKD), diabetes mellitus (DM), thyroid cancer or any other malignancies, pregnancy, and concurrent steroid therapy, or use of oral contraceptive pills were considered as exclusion criteria for patients having either of them during their regular follow-up.

Biochemical data

From each patient, 10 ml of blood was taken and put in a clot activator tube, centrifuged immediately, serum was separated and then frozen at -20 °C to be stored for analysis. TSH and anti-thyroid peroxidase antibody (TPO Ab) were analyzed by electrochemiluminescence (ECL) assay (Cobas e411 analyzer - Roche, Germany). The normal value for TSH is 0.27-4.2 μIU/ml and our measuring range was 0.005-100.0 μIU/ml. The normal value for TPO Ab is <34 IU/ml and our measuring range was 5-600 IU/ml. The patients were diagnosed with hypothyroidism based on a TSH level > 10 μIU/ml.

A total of 2667 TSH measurements (for new 1529, and known 1138 measurements) were made. A TSH of ≤ 4.2 μIU/ml was considered as controlled. As a result of the observed variable TSH levels in every patient over one and two years follow-up, we calculated the average TSH level for every patient for one and two years. The first TSH levels before initiation or adjustments of LT4 treatment were not included in the equation.

One year average TSH = (3^rd^ month TSH + 6^th^ month TSH + 9^th^ month TSH + 12^th^ month TSH) /4

Two years average TSH = (3^rd^ month TSH + 6^th^ month TSH + 9^th^ month TSH + 12^th^ month TSH + 15^th^ month TSH + 18^th^ month TSH + 21^st^ month TSH + 24^th^ month TSH) /8

An average TSH of ≤ 4.2 μIU/mL was considered as controlled.

Bodyweight measurement

For every patient in each visit, the BWT in kilogram (kg) and height in meter (m) were measured with bare feet and light clothes. Body mass index (BMI) was calculated by the formula of (BWT in kg/height m^2^). According to the World Health Organization, a value of a BMI of 30 or more defines obesity.

Statistical analysis

The data were analyzed by the Statistical Package for the Social Sciences (SPSS), version 26.0 (IBM SPSS Statistics, Armonk, NY). Categorical variables were summarized as numbers (N) and percentages (%). Continuous variables were summarized as mean ± standard deviations (M ± SD). The comparison of the controlled TSH frequencies through the visits over one and two years was made using Cochran’s Q test. A repeated-measures analysis of variance (ANOVA) with a Greenhouse-Geisser correction was used to compare the mean changes in the TSH levels and BWTs within visits over one year and two years. For in-between visits, TSH and BWTs comparisons, a post hoc test with Bonferroni correction was used. For all of the above comparisons, a *P*-value of < 0.05 defined statistical significance.

## Results

Table 1 summarizes the general characteristics of the 346 patients with hypothyroidism included in the study.

**Table 1:** General characteristics of the patients with hypothyroidism included in the study. BWT, bodyweight; BMI, body mass index; SD, standard deviation; TSH, thyroid-stimulating hormone; TPO Ab, anti-thyroid peroxidase antibodies.

	Mean ± SD or count/total (%)		*P-*value
	Known hypothyroidism (154)	New hypothyroidism (192)	
Women	143/154 (92.9)	158/192 (82.3)	0.004
Age (years)	47.1 ± 13.5	47.0 ± 12.6	0.92
BWT (kg)	79.9 ± 16.5	80.8 ± 17.5	0.60
BMI (kg/m^2^)	32.1 ± 6.4	31.6 ± 6.6	0.44
Obesity	97/154 (63.0)	113/192 (58.9)	0.43
TSH (μIU/mL) at enrollment	4.1 ± 3.1	43.07 ± 29.8	<0.0001
TSH ≤ 4.2 μIU/mL at enrollment	82/154 (53.2)	0/192 (0)	<0.0001
All TSH ≤ 4.2 μIU/mL after 3 months and through the study	662/984 (67.2)	739/1337 (55.2)	
TPO Ab (IU/mL)	381.8 ± 687.5	521.2 ± 924.7	0.19
TPO Ab ≥34 IU/mL	76/105 (72.4)	115/134 (85.8)	0.01
Goiter	30/154 (19.5)	38/192 (19.8)	0.94
Thyroidectomy	16/154 (10.4)	26/192 (13.5)	0.37

**Figure 2:**
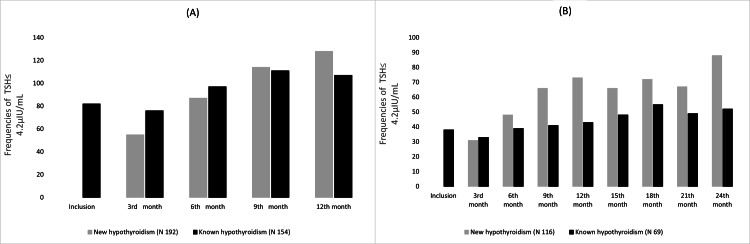
The frequencies of controlled TSH in patients with hypothyroidism, (A) over one-year follow-up, and (B) over two-year follow-up.

Over one and two years, there were significant differences in the frequencies of controlled TSH across visits for both patients with new and known hypothyroidism (Cochran’s Q test p < 0.0001 for new and known hypothyroidism over one and two years). There is an impression of an increasing degree of control over one year, as shown in Figures 2A-2B.

One year

**Figure 3:**
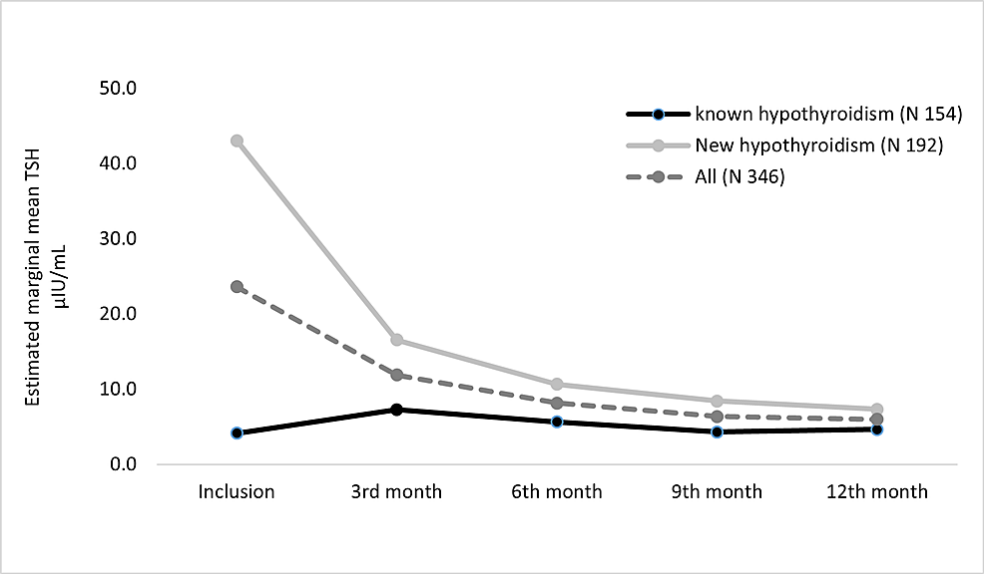
Estimated marginal mean of TSH over a one-year follow-up.

We evaluated 346 patients with hypothyroidism (known 154 and new 192) for changes in TSH levels and BWTs over one year. For all, new, and known hypothyroidism patients, the mean TSH level reduced significantly through the visits, (F(3.18, 1097.09) = 86.53, P < 0.0005), (F(2.98, 569.33) = 125.81, P < 0.0005), and (F(2.76, 423.37) = 4.90, P = 0.003, respectively). For all patients, post hoc test revealed that the TSH reductions were statistically significant between inclusion vs. all follow-up visits (P < 0.0005), 3^rd ^month visit vs. all follow-up visits (P < 0.0005), and no significant TSH changes through 6^th^, 9^th^, and 12^th ^month visits. For patients with new hypothyroidism, the post hoc test revealed a similar finding with significant TSH reductions in inclusion vs. all follow-up visits (P < 0.0005), 3^rd^ month visit vs. all follow-up visits (P < 0.0005), and no significant TSH changes through 6^th^, 9^th^, and 12^th^ months visits. However, for patients with known hypothyroidism, post hoc test revealed a significant increase in TSH level at 3^rd^ month visit from inclusion (P = 0.02), followed by a reduction in TSH level through the following visits, which were significant only in 3^rd^ vs. 9^th^ month visit (P = 0.008) and 3^rd^ vs. 12^th^ month visits (P = 0.04) (Figure 3).

**Figure 4:**
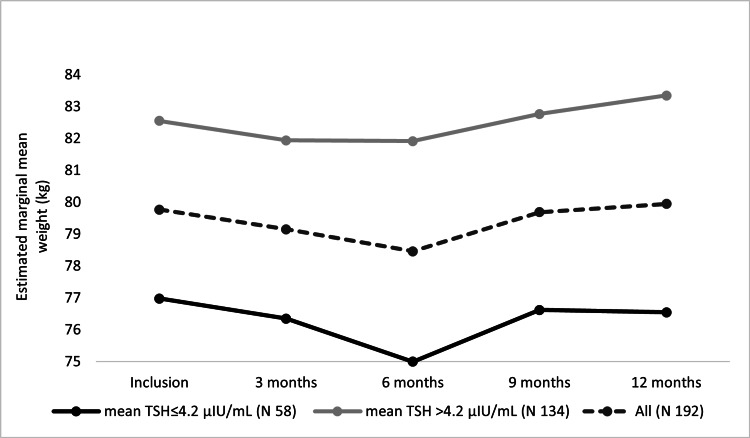
Estimated marginal mean BWTs of new hypothyroidism patients over one year.

A corresponding analysis was done for BWT changes through one year. As shown in Figure 4, for patients with new hypothyroidism, mean BWT changed statistically significantly through visits (F(2.29, 435.22) = 4.029, P = 0.01), with progressive BWT reduction from inclusion to 3^rd^ month to 6^th^ months visits (mean BWT reduction = 1.3 ± 0.5 kg at 6^th^ month visit). This reduction was followed by a progressive increase in the mean BWT from 6^th^ month to 9^th^ month to 12^th^ month visits, but with no significant changes on post hoc test (mean BWT increase = 0.18 ± 0.35 kg at 12^th^ month visit). On subgroups analysis, patients with an average TSH > 4.2 μIU/ml showed a similar statistically significant finding (F(2.41, 321.60) = 3.28, P = 0.03), and a statistically significant increase in the BWT on post hoc test between 3^rd^ month and 12^th^ month visits with P = 0.004 (mean BWT increase = 1.4 ± 0.38 kg from 3^rd^ to 12^th^ month visit, and mean BWT increase = 0.79 ± 0.41 kg at 12^th^ month visit). Analysis of the patients with average TSH ≤ 4.2 μIU/ml showed that the BWT changed non significantly but similarly (F(1.98, 112.85) = 2.27, P = 0.10), and no significant changes were noticed on post hoc test (mean BWT change = -0.43 ± 0.5 kg at 12^th^ month visit).

**Figure 5:**
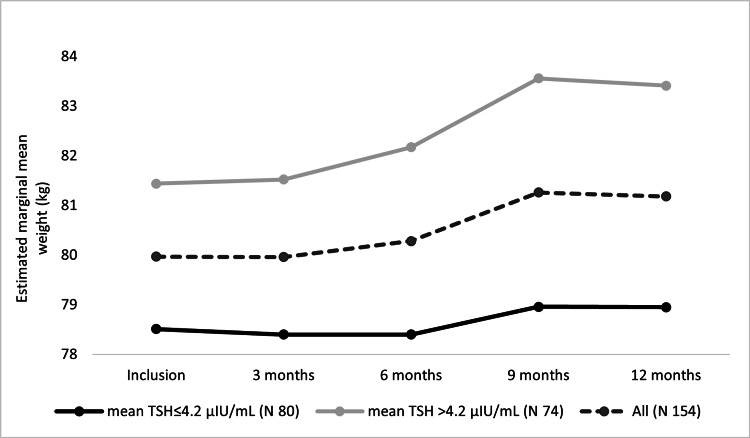
Estimated marginal mean BWTs of known hypothyroidism patients over one year.

For patients with known hypothyroidism, as shown in Figure 5, mean BWT changed statistically significantly through the visits (F(2.63, 400.35) = 7.00, P = 0.0002), with progressive BWT increase. On post hoc test, the BWT increased significantly at 9^th^ month visit in comparison to inclusion, 3^rd^, and 6^th^ month visits, (P = 0.01, 0.005, and 0.02, respectively), and also at 12^th^ month visit in comparison to 3^rd^ month visit (P = 0.03) (mean BWT increase = 1.20 ± 0.43 kg at 12^th^ month visit). On subgroups analysis, those patients with average TSH > 4.2 μIU/ml, a similar statistically significant finding was seen (F(2.56, 187.47) = 7.11, P = 0.0003), and a statistically significant increase in the BWT on post hoc test between 9^th^ month visit in comparison to inclusion and 3^rd^ month visit (P = 0.01, 0.01) and also at 12^th^ month visit in comparison to inclusion and 3^rd^ month visits, (P = 0.03, 0.01) (mean BWT increase = 1.97 ± 0.64 kg at 12^th^ month visit). Analysis of the patients with average TSH ≤ 4.2 μIU/ml showed that the BWT increased but non significantly (F(2.26, 178.52) = 0.87, P = 0.47), and no significant changes were detected on post hoc test (mean BWT increase = 0.44 ± 0.59 kg at 12^th^ month visit).

Two years

**Figure 6:**
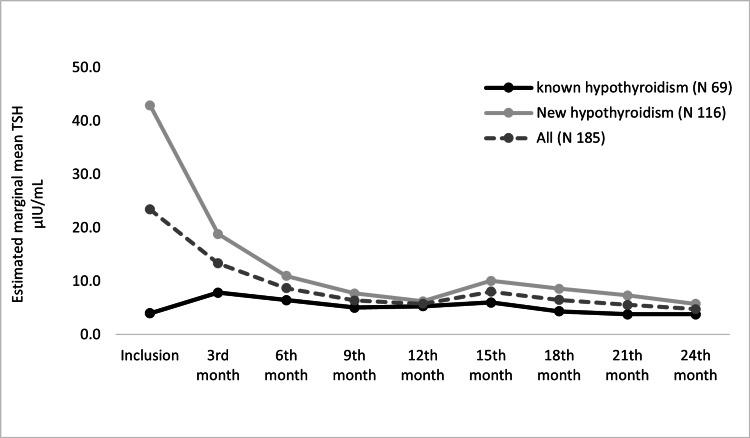
Estimated marginal mean of TSH over two years follow-up.

We evaluated 185 patients with hypothyroidism (known 69 and new 116) for changes in TSH levels and BWT over two years. As shown in Figure 6, for all patients and new hypothyroidism patients, the mean TSH level also reduced statistically significantly through visits over two years (F(5.26, 963.94) = 32.40, P < 0.0005 and F(4.96, 571.02) = 61.77, respectively; P < 0.0005). Post hoc test revealed that the significant changes were seen only through the first year, and no significant differences were seen through the second year visits (all: P > 0.05). For patients with known hypothyroidism, there was no significant change in the TSH level (F(3.71, 252.78) = 2.14, P = 0.08).

**Figure 7:**
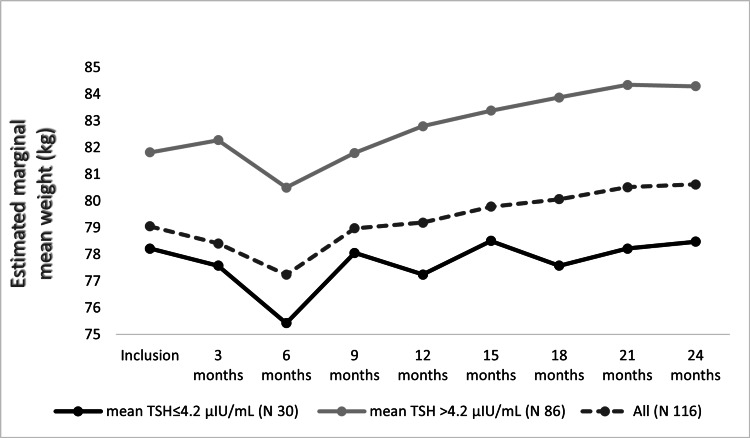
Estimated marginal mean BWTs of new hypothyroidism patients over two years.

For the patients with new hypothyroidism, a corresponding analysis for BWT changes over two years follow-up was done (Figure 7). The BWT increased significantly through visits (F(2.97, 339.09) = 5.09, P = 0.002) (mean BWT increase = 1.57 ± 0.80 kg at 24^th^ month visit). But on the post hoc test, no significant BWT changes were detected between visits. On subgroups analysis, patients with an average TSH > 4.2 μIU/ml witnessed a similar statistically significant increase in the BWT (F(3.10, 263.89) = 9.08, P < 0.0005). On post hos test, the BWT was significantly increased at 24^th^ month visit in comparison to 3^rd^ (mean BWT increase = 3.02 ± 0.77 kg), 6^th^ (mean BWT increase = 3.80 ± 1.03 kg), and 9^th^ (mean BWT increase = 2.50 ± 0.70 kg) months visits, (P = 0.007, 0.015, 0.023, respectively). Analysis of the patients with average TSH ≤ 4.2 μIU/ml showed that the BWT changed non significantly (F(2.57, 77.20) = 1.10, P = 0.34). Post hoc test also revealed no significant BWT changes between visits (mean BWT increase = 0.25 ± 1.35 kg at 24^th^ month visit).

**Figure 8:**
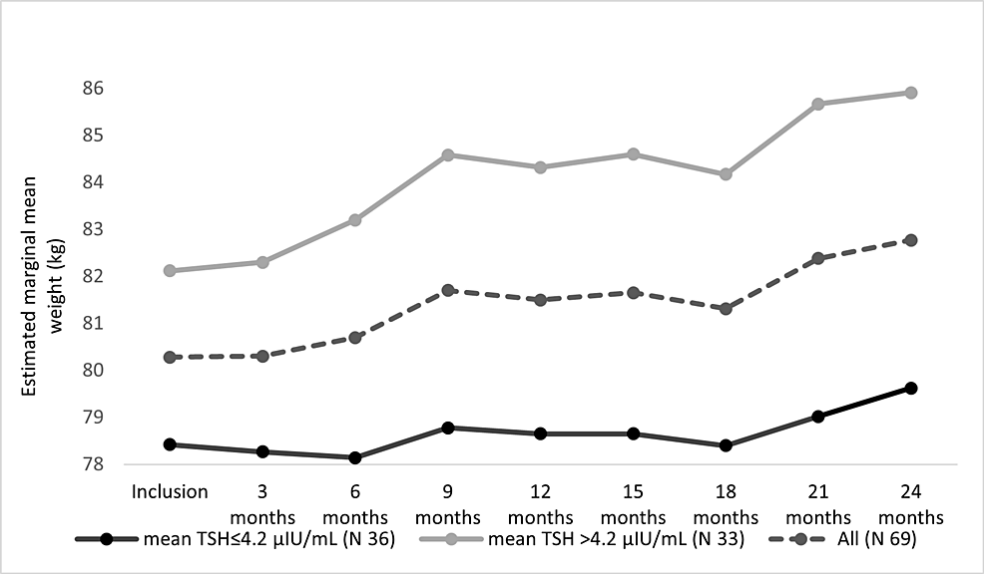
Estimated marginal mean BWTs of known hypothyroidism patients over two years.

For patients with known hypothyroidism, the BWT increased significantly through the visits over two years (F(2.81, 188.63) = 5.27, P = 0.002), as shown in Figure 8. Post hoc test revealed no significant BWT changes between visits except between 24^th^ and 3^rd^ months visits (mean BWT increase = 2.4 ± 0.71 kg; P = 0.03). On subgroups analysis, patients with an average TSH > 4.2 μIU/ml showed a similar statistically significant increase in the BWT (F(2.35, 77.56) = 4.67, P = 0.009) (mean BWT increase = 3.78 ± 1.26 kg at 24^th^ month visit). For patients with average TSH ≤ 4.2 μIU/ml, the analysis showed that the BWT changed non significantly (F(3.94, 134.22) = 1.14, P = 0.34) (mean BWT increase = 1.20 ± 0.77 kg at 24^th^ month visit). For each group, the post hoc test revealed no significant BWT changes between visits (mean BWT increase = 0.25 ± 1.35 kg at 24^th^ month visit).

## Discussion

During the two years of follow-up in this study, the BWT changes had different significance levels with the TSH, and the TSH could not predict the BWT changes during the study or vice versa. A significant association between the BWT changes and the TSH level was reported in the first six months of the POUNDS LOST trial, but not for the overall 24 month period [6]. In comparison, Knudsen et al. demonstrated a significant positive association between serum TSH and BWT gain during a more extended period (five years) but not six months [7]. Similar findings of no significance during the different periods during the same study were observed by Fox et al. [8], Svare et al. [9], and Bjergved et al. [10].

It is difficult to ascertain the exact direction and etiology of association between BMI and TSH, whether this association is negative [11,12], positive [7-10,13-15], or not present at all [6,16-21].

The low metabolic rate expanded water and fat mass; excess accumulation of water-binding glycosaminoglycans in patients with longstanding and severe hypothyroidism might contribute to the BWT changes towards more overweight, with different degrees of association [6,7,12,22,23]. Loss of BWT and increase in resting energy expenditure (REE) in individuals with hypothyroidism after reestablishing euthyroidism with LT4 therapy might be explained by excretion of excess water and reduced lean body mass rather than a reduction in the fat mass [12,23].

The discrepant findings of the association between thyroid function and BMI might be attributed to the difference in participants' characteristics, like age, gender, the bidirectional changes between BMI and TSH, and the different durations for the follow-up.

Even within its normal reference ranges, small changes in TSH levels may significantly affect BWT, especially with LT4 therapy [7]. This is attributable to the minor alterations in the regulation of REE and physical activity, resulting in a mismatch between energy intake and energy expenditure, with uncertain effect on final BMI at different severities of hypothyroidism, for variable durations and different responses [1,2,7,17,19,20,23,24].

BWT reduction, whether intentional or in the course of a catabolic state, may reduce TSH, although with uncertain significance [7,25,26], with a relevant role of changes in leptin levels during different stages of BWT changes [6]. Obese individuals often present with transient hyperthyrotropinemia as a consequence, rather than the cause, of BWT excess [27,28]. However, the changes in thyroid hormones are controversial or bidirectional in obesity in a complicated way [2,5], regarding which one is the primary or the secondary event, the alterations in thyroid function, or the increase in BMI [20].

The study's retrospective, and so it can not show causality between BWT changes and the TSH during the study period. The associations between BMI and TSH found in various studies did not necessarily imply a causal relationship. TSH and BMI could be affected by many factors other than each other.

Three large community-based studies on 19371 individuals demonstrated that the TSH changes were significantly associated with the subsequent BWT changes and not vice versa [8-10].

We studied the BWT changes on different TSH concentrations and showed mixed results of different levels of significance. In Díez et al., the association between serum TSH and obesity was only significant in patients with serum TSH > 3.6 μIU/mL. Individuals with serum TSH levels in the highest tertile had the highest BMI values [14]. Boeving et al. showed a lack of correlation between the degree of TSH suppression by LT4 therapy and BMI regardless of the LT4 therapy and at any level of TSH between 0.4 and 4 μIU/mL [16]. Karmisholt et al. showed that individuals with TSH levels in the upper ranges have higher BMI, and those with TSH in lower ranges had a lower BMI [23].

There was a trend for the BWT changes in the enrolled patients with hypothyroidism toward modest initial reduction after initiation of LT4 therapy, then gradual step-up of BWT during the next 24 months of follow-up. Our findings were similar to Hoogwerf et al. and Weinreb et al., which demonstrated an initial reduction in BWT followed by a return to baseline BWT after one year, with a step-up pattern [3,21]. However, the exact mechanism behind the degree of BWT change with thyroid dysfunction and the LT4 effect was poorly understood [5]. The significant step-up BWT gain suggests that mild iatrogenic hyperthyroidism does not promote BWT loss or prevent aging-related BWT gain [29].

The early BWT loss following LT4 therapy to the pre-hypothyroidism level reflected the loss of myxedematous tissue [3,23], while the total body energy equilibrium is maintained. Karmisholt et al. attributed this reduction further to the reduced capacity of renal free-water excretion, increased antidiuretic hormone level, and increased amount in tissues of glycosaminoglycans, which have a large water-binding capacity [23].

There was an overall BWT gain of 3.02 ± 0.77 kg and 3.78 ± 1.26 kg for patients with new and known hypothyroidism, respectively, after achieving euthyroidism within the two years of the follow-up. The Tromsø study revealed a mean BWT gain of 2.8 kg within the seven years of the study [15]. We did not explain the difference between the studies and why the patients in our study had more BWT gain within two years more than what was achieved in seven years in Tromsø's.

In this study, most of the patients had obesity from the start. Finally, they either maintained or gained more BWT, indicating that obesity will continue as an important complaint in those patients. This finding had been observed in many previous studies [11,30].

We could not include the measurements of free thyroxine and triiodothyronine in our analysis, which could add more information about the thyroid status of the patients because over the years, we have different platforms of different reference ranges and because these measurements were not available for all patients during their follow-up. The inaccessibility to the dual x-ray absorptiometry made us unable to verify the exact nature of loss in lean body mass. We could not evaluate the concurrent nonthyroidal illnesses during the follow-up, which affected both the ultimate BWT and TSH of patients with hypothyroidism on LT4 therapy. We have no data about energy balance through dietary factors and physical activity, which are essential covariates for thyroid function and ultimate BWT changes.

## Conclusions

In contrast to what is believed, adequate treatment with LT4 does not associate with BWT reduction. Instead, either the patient maintained the same BWT or continued to gain more BWT. The exact association between TSH and BMI could not be confirmed through the study.
